# A community intervention for behaviour modification: an experience to control cardiovascular diseases in Yogyakarta, Indonesia

**DOI:** 10.1186/1471-2458-13-1043

**Published:** 2013-11-04

**Authors:** Fatwa Sari Tetra Dewi, Hans Stenlund, V Utari Marlinawati, Ann Öhman, Lars Weinehall

**Affiliations:** 1 Public Health Division, Faculty of Medicine, Gadjah Mada University, Yogyakarta, Indonesia; 2 Epidemiology and Global Health, Department of Public Health and Clinical Medicine, Umeå University, Umeå, Sweden; 3 Umeå Center for Global Health Research, Umeå University, Umeå, Sweden; 4 Center for Health and Nutrition Research Laboratory, Faculty of Medicine, Gadjah Mada University, Yogyakarta, Indonesia; 5 Umeå Centre for Gender Studies, Umeå University, Umeå, Sweden; 6 Ageing and Living Conditions, CPS, Umeå University, Umeå, Sweden

**Keywords:** CVD prevention, Community-based intervention, Community-empowerment, Primary prevention

## Abstract

**Background:**

Non-communicable Disease (NCD) is increasingly burdening developing countries including Indonesia. However only a few intervention studies on NCD control in developing countries are reported. This study aims to report experiences from the development of a community-based pilot intervention to prevent cardiovascular disease (CVD), as initial part of a future extended PRORIVA program (Program to Reduce Cardiovascular Disease Risk Factors in Yogyakarta, Indonesia) in an urban area within Jogjakarta, Indonesia.

**Methods:**

The study is quasi-experimental and based on a mixed design involving both quantitative and qualitative methods. Four communities were selected as intervention areas and one community was selected as a referent area. A community-empowerment approach was utilized to motivate community to develop health promotion activities. Data on knowledge and attitudes with regard to CVD risk factors, smoking, physical inactivity, and fruit and vegetable were collected using the WHO STEPwise questionnaire. 980 people in the intervention areas and 151 people in the referent area participated in the pre-test. In the post-test 883 respondents were re-measured from the intervention areas and 144 respondents from the referent area. The qualitative data were collected using written meeting records (80), facilitator reports (5), free-listing (112) and in-depth interviews (4). Those data were analysed to contribute a deeper understanding of how the population perceived the intervention.

**Results:**

Frequency and participation rates of activities were higher in the low socioeconomic status (SES) communities than in the high SES communities (40 and 13 activities respectively). The proportion of having high knowledge increased significantly from 56% to 70% among men in the intervention communities. The qualitative study shows that respondents thought PRORIVA improved their awareness of CVD and encouraged them to experiment healthier behaviours. PRORIVA was perceived as a useful program and was expected for the continuation. Citizens of low SES communities thought PRORIVA was a “cheerful” program.

**Conclusion:**

A community-empowerment approach can encourage community participation which in turn may improve the citizen’s knowledge of the danger impact of CVD. Thus, a bottom-up approach may improve citizens’ acceptance of a program, and be a feasible way to prevent and control CVD in urban communities within a low income country.

## Background

An estimated 60% of global deaths and 80% of all deaths in developing countries are due to non-communicable diseases (NCD), and cardiovascular disease (CVD) is responsible for half of these [1]. In 2008, CVD is responsible for 17.3% million deaths per year, and nearly 10% of global disease burden is attributed to CVD [2]. Most initiatives to control CVD have occurred in high income countries [2] and had some success in reducing CVD prevalence. In low and middle income countries, few studies to control CVD have been implemented, and CVD prevalence continues to increase [3].

Compared to the CVD control programs in low and middle income countries that focus mainly on secondary prevention [4,5], programs in high income countries are more comprehensive and focus on both primary and secondary prevention. Programs in high income countries focus on primary prevention to reduce CVD risk factors through increased awareness of healthy lifestyles, and secondary prevention through early detection, and improved treatment [4]. In contrast, low and middle income countries programs address primary prevention through reduction of CVD risk factors are rare [6].

As a number of unhealthy behaviours have been identified as the risk factors for CVD (smoking, unhealthy diet, sedentary lifestyle, and excess alcohol consumption), preventing CVD asks for changing these behaviour [7]. Behaviour is both depending on individual choices and social support [8]. Behaviour changing initiatives both focusing on individuals, population and social environment requires a long time to be able to show results. They are therefore costly. This reality is an important limitation for community-based intervention program evaluations in low and middle income countries. One possible solution to overcome this limitations is a process evaluation [9] where its indicators might be relevant even if data on biomedical outcomes are lacking [10].

Therefore, to control CVD in low and middle income country, investigations of a community-based primary-prevention of CVD risk factors are needed. The PRORIVA Program (Program to reduce cardiovascular disease) is an urban community-based intervention study in Yogyakarta city, Indonesia. It is aimed at primary prevention of CVD through behaviour modification at the individual and community levels.

The social ecological model explains that behaviours are determined by multiple levels of influence of others at intrapersonal, interpersonal, organizational, community, and policy levels [8]. To modify individual behaviour it is therefore necessary to perform community interventions by involving communities in defining the behavioural problem, seeking potential solutions, and executing the solutions. Under this model, it is expected that the higher the community participation, the greater the potential for behavioural changes. This community empowerment strategy is aimed at community-wide behaviour modification, uses a community organizing approach, and has been applied in HIV primary prevention [11]. In this paper, the approach was utilized to motivate participation. Further details of the PRORIVA program are reported elsewhere [12].

The aim of this paper is to report a process evaluation and a short term evaluation of a small-scale pilot intervention (stage 4) in four Indonesian urban communities.

## Methods

### The study design

**Figure 1:**
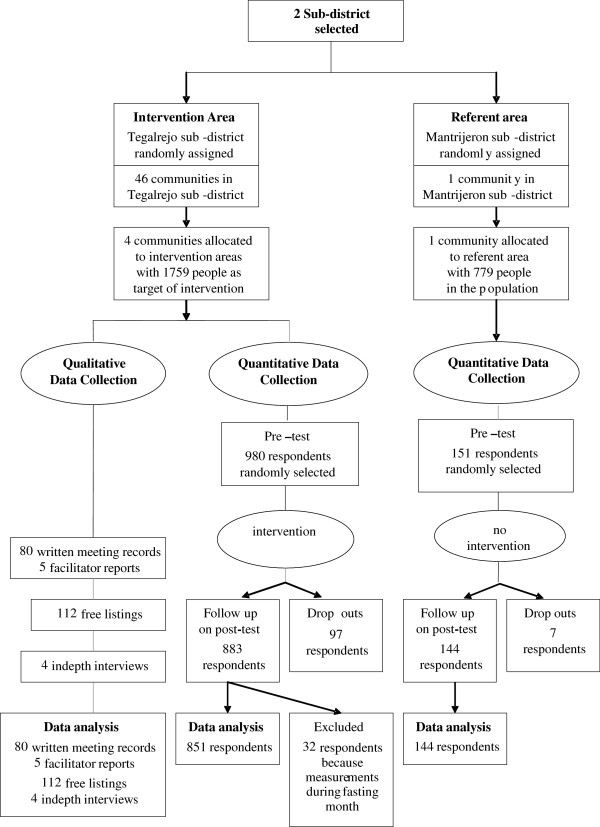
The process of data collection in the intervention and referent communities.

The study combines quantitative and qualitative methods based on the priority-sequence model developed by Morgan [13]. Based on priority of decisions, a quantitative approach was decided to be the principal methodology for data collection and consequently qualitative methods were complementary. Based on sequence of decisions, we first conducted the quantitative part of the study and followed this with qualitative methods to deepen the understanding of the quantitative results. The quantitative part describes the participation level and appraises the program effects by comparing healthy behaviours before (pre-test) and after (post-test) the intervention. The qualitative study aims to understand people’s motives and responses to the intervention using in-depth interviews, free-listing, and written meeting records [14], Figure 1.

#### The quantitative study design

A quasi-experimental study design [15] was applied as part of the PRORIVA study. Two sub-districts, Tegalrejo and Mantrijeron, were included in the study. These two sub-districts were selected as they are all in urban areas, have similar average of ages, income, similar mass media exposure, however geographically separated (± 10 km) to minimize cross-contamination bias. The median of age was 26.0 year in Tegalrejo and 26.2 year in Mantrijeron. The men/women proportion in both Tegalrejo and Mantrijeron was 0.97. After random assignment, Tegalrejo sub-district was selected as the intervention area and Mantrijeron sub-district served as the referent area. In the intervention sub-district, out of 46 communities, two communities were selected representing typical high socioeconomic (SES) communities and two representing low SES communities, according to the poverty registry [16]. In the high socioeconomic (SES) communities, median of age 26.6, men/women = 0.97 and the low SES communities, median of age 26.6, men/women = 0.96 [16]. Resource limitation enabled only to include one referent community which has similar prevalence of CVD risk factors as the four intervention communities but represented as a typical middle SES community, median of age 26.7, men/women = 0.96. A health promotion program targeted the entire population aged 15 to 75 years (1759 people) in the intervention communities, Figure 1. As an initiation program, PRORIVA focused on adult whom their lifestyle has been set up. Leaflets on health promotion were distributed to the population both in the intervention and referent communities one month after the intervention to make them unaware whether they were in the intervention or referent community.

#### The qualitative study design

The qualitative study reports PRORIVA as a case that consists of low and high SES communities that were constantly compared [17]. Qualitative content analysis as described by Graneheim & Lundman was used and focused on the manifest content [18] in three domains (ie, motives for participation, behavioural change, and perceived benefit of the PRORIVA program). In conducting the analysis, the first and third authors read through the data to understand the text as a whole, identified and abstracted each meaning unit, and labelled the meaning units as codes. The codes were constantly compared for differences and similarities between the low and high SES communities. The codes were then organized into the three domains to describe participant impressions of the PRORIVA program.

Trustworthiness of the qualitative part was ensured by using three different techniques: 1) prolonged engagement, 2) peer-debriefing, and 3) triangulation of researchers [19]. The first author and the PRORIVA team convinced the community leadership to participate in the program. The first author was present during community meetings, some group activities, and the mass action intervention. The third author was well-informed about the PRORIVA program and given the raw qualitative data for reading and criticism of data interpretation. The draft results of data analyses were presented to the third author and discussed for possible interpretations. Finally, the fourth and fifth authors confirmed or modified descriptions of the three domains.

### The PRORIVA small scale intervention

The PRORIVA small-scale intervention started in September 2006 and lasted for seven months. PRORIVA underlined the process of motivating community participation. The citizens were encouraged to participate in identification of CVD as a health problem, and to give input in tailoring the program. The PRORIVA intervention was designed both on the results of a baseline survey and of a qualitative study from 2004 [12], where the baseline survey measured CVD risk factors in order to establish priorities in the target population and the qualitative study assessed perceptions about CVD, its risk factors, and its prevention within the local context.

The PRORIVA intervention included an implementation process in five phases: 1) building trust with the community; 2) raising community awareness; 3) program development; 4) community organizing; and 5) initiation of maintenance. Specific activities were developed for each phase, Table 1, addressing smoking, physical inactivity and low fruit and vegetable consumption, as these represented three major behaviours shown to be strongly related to CVD risk [20].

**Table 1:** Phases and activities in the PRORIVA small-scale intervention

**Community intervention phases and activities**	**Description of activities**
Building trust	
Community leader meeting	Meetings for community leaders conducted separately for certain level of community leaders. Usually discuss how to solve the problem in their community and to socialize program
Public awareness	
Regular Public Meeting	A once a month meeting conducted separately among fathers and mothers usually discuss community problems and socialize program. In these meetings we communicated messages in every stage of intervention including health education and a forum to arise decision on non-smoking meeting.
Program development	
Team works meetings	A once a week meeting involving PRORIVA team, key person and health workers to design, implement and monitor the program
Community Organizing	
CVD information posts	Posts where people can access CVD risk factors screening, health counseling, and necessary referral to health service. These posts open regularly about 4 hours twice a week.
Sunday Morning Walking	Walking together voluntarily for all people every Sunday after morning praying. Start with a short health speech, risk factors screening, walking for 30 minutes, ended with healthy refreshment.
Weekly Exercise Group	Aerobic dancing groups, conducted once a week with local instructor, and mostly attended by mothers. Started with short health speech, risk factors screening and dancing for 30 minutes.
Initiation of maintenance	
Cooking Competitions	Competition between groups of ten-household-mothers to provide healthy cooking from certain raw materials, for example from soya.
Aerobic Dancing Competitions	Competitions between self-arranged group consist of 4–10 people to perform aerobic dancing
Health Speech Competitions	Competitions between health workers to deliver health message on CVD prevention
Healthy Walking Competitions	Competitions for all people to perform moderate walking.
Public festival	A public feast conducted to acknowledge the volunteers working for PRORIVA, and to announce the champions of competitions

In the 1) trust-building and 2) raising awareness phase, the actions were performed simultaneously through lobbying, presentations, and self-identification of CVD cases among relatives. Initially, community leaders were involved and then citizens were included.

In the 3) program development phase some working teams were established in each community consisting of one facilitator from the study group, one local contact person, and some health workers from each community. This team shared basic information about CVD control, reached agreement on their roles and responsibilities and developed the program design together.

In the 4) community organizing phase, the community members were invited to participate in activities agreed upon by the working team. Most activities were new initiatives (CVD Information Post, Cooking Competitions, Aerobic Dancing, Healthy Walking, and Health Speeches Competitions), while others were revitalization of previously existing activities (Regular Public Meetings, Sunday Morning Walking, and Weekly Exercise Groups).

Different media products such as leaflets, posters, booklets, flipcharts, books, warning signs, food models, and audio-visual aids, were prepared and pre-tested to support the activities. The messages presented in the media were adapted to community demand and were pre-tested, generally included ‘What is CVD?’, ‘How dangerous is the disease?’, ‘What is the cause of CVD?’, and ‘What can be done to prevent the disease’. These actions lasted up to four months. Lastly, during phase 5) preparation of maintenance phase, two months after the community organization action was begun, gatherings were held to evaluate the activities and take steps to support program sustainability. A network was built that consisted of primary health care providers and health officials. These network members gradually took over the responsibility to facilitate the program while the personnel from study group gradually diminished their own roles.

### The quantitative data collection

A sample of respondents were selected both in the intervention and referent population, the respondents were pre-tested and post-tested before and after the intervention. The inclusion criteria for the sample were aged 15–75 years, were able to stand straight, were living in the research area min 6 months, and agreed to participate. The instrument for the pre and post-test was based on the STEPwise questionnaire for non-communicable disease risk factor surveillance which incorporated questions about behaviour (smoking habits and fruit and vegetable intake) and physical measurements (height, weight, blood pressure) [21]. Additional questions about knowledge and attitudes toward CVD were asked. Data were collected by trained surveyors under the coordination of a supervisor. Supervisor checked the completeness of data collection and re-interviewing 5% of respondents to check the validity of data. Minutes of the activities were reviewed to describe average participation by number of participants, types of health promotion actions, and types of participants.

Behaviour patterns were analysed before and after the intervention. Knowledge about CVD was measured with eight questions. Low knowledge was defined as a value below the mean value of 6, and high knowledge was defined as 6 or higher. Attitudes toward CVD were measured with 12 questions and individuals were scored from 12 to 60. Respondent attitudes were classified as negative (more disagreement about prevention of CVD through risk factors modification) if the total attitude score was below the mean (<36) and positive for scores 36 or higher [22]. Respondents were classified as smokers if they reported smoking at least one cigarette per day. Respondents were classified as having low fruit and vegetable intake if they ate <4.5 portions per day [23]. A total activity time of <150 min per week was regarded as physically inactivity according to WHO criteria [24]. A community was defined as low SES if more than 70% of households living below the poverty line, and as high SES if less than 20% of households living below the poverty line according to household’s income, ownership, and expenditures defined by the Government of Yogyakarta Municipality [16].

### Statistics

The proportions of health behaviours at the pre-test were analysed using Chi-square tests between respondents of the intervention and referent communities. Differences in proportions of health behaviours between the pre- and post-test were analysed using McNemar tests. Odds ratios (OR) were used to estimate the risk of having unhealthy behaviours for respondents in the intervention community compared to those in the referent community. The ORs were calculated using logistic regression and controlled for pre-test behaviours in order to account the behaviour at pre-test as one of the influencing variables of behaviour at post-test. Statistical procedures were performed using STATA version 11 (StataCorp LP, College Station, Texas, USA), and the significance levels were set at *p* <0.05.

### The qualitative data collection

During the intervention, activities were documented regularly by meeting minutes and facilitator reports about who participated and how the activities were carried out, why the study can be regarded as process evaluation. To know whether the target audiences recognize the PRORIVA, a free-listing was filled-out after the post-test in the intervention communities. Informants were selected employed stratified purposeful [17] sampling from subgroups of community leaders, health workers, and citizens who came on some community meetings and visited a local shop. In the free-listing data collection technique, participants listed all items related to questions about what activities were known components in the PRORIVA program, perception of the PRORIVA in general, and whether the respondents had any suggestions for program improvement. Finally, further elaboration of informant free-listing was sought through interviews with four participants who were selected on the basis of the rich information they wrote in the free-listing. After four additional individual interviews, data collection was finished.

The ethics committee at Gadjah Mada University and the Government of Yogyakarta Municipality approved this study, reference number KE/FK/209/EC. After the necessary information and explanations were given, written informed consent was obtained from each participant before quantitative data collection and a verbal informed consent was obtained before qualitative data collection.

## Results

### The quantitative study

The target population of the study areas was 2538 people. Of those, a sample of 1131 respondents was randomly selected to be included at the pre-test. Nine hundred and ninety five respondents participated at both pre- and post-test; 104 dropped out at the post-test; 32 were excluded because their data were collected during a month of fasting, Figure 1.

The pre-test was not carried out simultaneously in all study areas, because of a delay in community acceptance. In each community the pre-test was finished before the intervention was performed to prevent the intervention to infer with the pre-test.

Table 2 illustrates that the number of activities events in low SES communities (40 times) were three times as frequent as those in the high SES communities (13 times), although the average participation was similar between the low and the high SES communities. More people participated on large group activities in the low SES communities than in the high SES communities. In addition, people in the low SES communities participated more frequently in the program within all health promotion actions compared to those in the high SES communities. The leader and other community members had high participation rates in the low SES communities; in high SES communities only the leaders showed a high participation rate.

**Table 2:** Characteristics of participants in activities in local communities with low and high socioeconomic status

**Activities by characteristics**	**Low SES**		**High SES**	
	**Number of events**	Average participation* (%)	**Number of events**	Average participation* (%)
All activities	40	50	13	51
Activities by number of eligible participants				
Small group (≤20 people)	17	56	11	59
Large group (21–50 people)	20	48	2	10
Mass (>50 people)	3	29	0	0
Activities by health promotion action				
Trust-building	2	100	2	100
Raising awareness	1	83	4	35
Developing programs	3	67	5	61
Community organizing	29	47	1	36
Preparation for maintenance	5	27	1	17
Activities by type of participants				
Leaders	1	83	2	100
Health workers	11	75	5	61
Residents				
Men only	6	65	4	35
Women only	13	34	1	4
Both sexes	9	29	1	17

$*=\frac{\sum \left(\frac{\mathrm{Number}\;\mathrm{of}\;\mathrm{participants}\;\mathrm{participated}}{\mathrm{Number}\;\mathrm{of}\;\mathrm{eligible}\;\mathrm{participants}}\times 100\%\right)}{\mathrm{Number}\;\mathrm{of}\;\mathrm{events}}$

Separate analyses were performed for men and women. At pre-test, there were no significant differences in healthy behaviors (p = 0.053), even if knowledge among men was 69% in the referent area compared to 59% in the intervention area. At post-test, using logistic regression analysis, there was no significant difference in knowledge between men in the referent and intervention areas after adjusting for knowledge at pre-test. However, within the intervention areas, knowledge increased significantly from 59% to 70% (p <0.0001), Table 3.

**Table 3:** Proportion of behaviours related to cardiovascular disease at pre-test and post-test among men and women

**Health behaviour**	**Type of community**^**a**^	**Men (n = 448)**		**Women (n = 547)**			
		**Pre-test (%)**	**Post-test (%)**	***p*-value (McNemar)**	**Pre-test (%)**	**Post-test (%)**	***p*-value (McNemar)**
High knowledge^b^	I	56	70	0.000	60	75	0.000
	R	69	80	0.167	58	76	0.006
Positive attitude^c^	I	26	28	0.624	27	31	0.191
	R	26	28	1.000	31	34	0.860
Non smoker^d^	I	55	53	0.302	100	100	N.A.
	R	52	56	0.727	100	99	N.A.
Physically active^e^	I	55	51	0.388	51	48	0.402
	R	48	61	0.169	52	39	0.090
Sufficient fruit and vegetable intake^f^	I	13	13	0.826	12	17	0.033
	R	10	24	0.146	9	23	0.012

^a^I = Intervention communities, number of respondents was 851; R = Referent communities, number of respondents was 144.

^b^High knowledge defined as a total knowledge score ≥6 (the mean score).

^c^Positive attitude as a total attitude score ≥36 (the mean score); positive attitude means more agreeable to behaviour change to prevent CVD.

^d^Smoker was defined as smoking at least one cigarette per day.

^e^Physically active was defined as total activity time of ≥150 minutes per week.

^f^Sufficient fruit and vegetable intake was defined as eating ≥4.5 portions of fruit and/or vegetables per day.

### The qualitative study

One hundred and twelve informants participated in the free-listing procedure and four informants participated in in-depth-interviews. Five facilitator reports and 80 records from meeting minutes and activities were analysed. Based on these empirical data, we report content from the qualitative part within the three domains: a) motives for participation, b) behaviour change, and c) perceived benefit of the PRORIVA program components, Table 4.

**Table 4:** People’s motives and responses to Proriva

**Domains**	**Codes**	**Quotations**
**The motives for participation**	**Sub-group: Low SES communities**	
	Inconvenient feeling	“…what a pity if nobody shows up when an external party (the PRORIVA team) has come up with some activities, spends some money, and spends some time.… (a woman, citizen of a low SES community)
	Economic constrain	“To eat more portions of fruit--rarely we do it because of our economic conditions.” (a man, citizen of a low SES community)
	**Sub-group: High SES communities**	
	Individual activities preference	“It is difficult to arrange a citizens’ meeting here, even when we need to vote for the head of this community, it was only one person and me (who showed up at the meeting). They sent a message that they would pay some money instead (to support the community rather than to attend) and they just asked me to be the head”. (a man, community leader of a high SES community)
	More sophisticated program	“We request that this education be performed routinely….It should be improved if possible, for example with an *EKG* (Electrocardiograph) examination or other early detection activities.” (a man, citizen of a high SES community)
**Behavior change**	**Sub-group: Low SES communities**	
	Commonly reported: eating more vegetables	“…we were more careful to select healthier food and avoid the fatty foods”. (a woman, citizen of a low SES community).
	Rarely reported: Changing smoking habit	“Actually, I don’t want to let my husband continue smoking, but there’s no choice. He has been a smoker for long time….I realize how important it is to have exercise after participating in the exercise group and Sunday morning walking.” (a woman, citizen of a low SES community)
	**Sub-group: High SES communities**	
	Frequently adopted: becoming more physically active	“It is good (the PRORIVA), in fact the exercise group for women was routinely conducted in these communities. Exercise is a fun activity for all of us. It is cheap and easy.” (a woman, citizen of a high SES community)
	Least reported: changing smoking habits	“It is difficult to quit smoking during a community meeting where so many others are still smoking.” (a man, citizen of a high SES community)
**Perceived benefits of the program**	**Sub-group: Low SES communities (lay people)**	
	Exciting program	“…and then we conducted a gymnastics competition….there were three people who won from our neighbourhood. In short, it was such a happy time; in short, we want the program again.” (a woman, citizen of a low SES community)
	Demand for continuation	“It is a positive program, (and will be more so) if it is continued.” (a man, citizen of low a SES community)
	**Sub-group: Low SES (Health workers)**	
	Improve the capacity to deliver messages	“We feel that we improved our knowledge and it is our duty to disseminate those messages. If there is a (person from the) PRORIVA team, we will feel more motivated.”
	A refreshing program	“The aerobic dancing was relaxing. OK…, like *Poco-poco* (a kind of dancing from eastern Indonesia). It was so motivating. If it was only routine activity like it used to be, it was boring.” (a woman, health worker of a low SES community)
	**Sub-group: High SES communities (Lay people)**	
	A good program	“In this community we support the activities such as healthy heart group exercise, and preparation of nutritious meals rich in fibre.” (a woman, citizen of a low SES community)
	**Sub-group: High SES (Health workers)**	
	Uncomfortable with their role	“I don’t feel confident to deliver (information) to the citizens during citizen meetings. I would like to (deliver information), however I was scared of being perceived as looking like a very knowledgeable person.” (a woman, health worker in a high SES community)

### The motives for participation

#### Low SES communities

In the low SES communities, citizens may have felt it rude not to participate in activities held by others who sacrificed for their communities. This may explain reports that it was “inconvenient” not to participate in activities supported by the PRORIVA team or to invitations from health workers.

As confirmed in interviews, barriers to adoption of healthier behaviours were lack of fitness with daily activities, a shortage of local instructors for the exercise groups, and economic constraints within the community. This was especially the case for not buying more fruit. Men and women chose walking as the main physical activity. Young women preferred the exercise group. After the program ended, walking continued among individuals, and the exercise group continued with a smaller number of participants.

#### High SES communities

In contrast, informants in the high SES communities were hardly involved or attended the program. This was not because they ignored their health, but probably because they preferred individual health promotion activities even though those required further demands on their economic resources. A confession from a high SES community leader reflected how the citizens disliked community programs. One possible barrier was the lack of a more sophisticated program, such as provision of early detection for CVD, and not just behaviour modifications.

### Behaviour change

#### Low SES communities

During the free-listing procedure in low SES communities, the most commonly reported health behaviour modification was eating more vegetables, Table 4. Eating more fruit, being more physically active, or not being a passive smoker was also commonly reported. The least reported behaviour was changing a smoking habit. Only two men were concerned about smoking. One man wrote that he should quit smoking and the other wrote that he should reduce the number of cigarettes he smoked.

#### High SES communities

In the high SES communities, respondents claimed that becoming more physically active and avoiding passive smoking were frequently adopted. Also commonly reported was eating more vegetables. The least reported changes were eating more fruit and changing smoking habits.

### The perceived benefits of the PRORIVA program

The perceived benefits of the PRORIVA program were divided into a citizen point of view and a health worker point of view, Table 4. Perceived benefit refers to how well the program was able to overcome the threat of disease, to solve their health problems, and to ease their lives.

#### Low SES communities (Lay people)

From the citizen point of view, the PRORIVA program was perceived as raising their awareness of the CVD threat. They demanded continuation of the program. They felt the small gifts drew their curiosity for participation in the action programs. Most of the low SES community informants who participated in the free-listing considered the PRORIVA activities to be impressive. They claimed PRORIVA was “exciting”, “cheerful”, and they felt “sadness after the program finished”. Men reflected that the program was good; women additionally thought that not only was the program good, but they felt that they actively supported the program.

#### Low SES communities (health workers)

The health workers believed that they improved their skills at delivering messages regarding CVD prevention. They requested the program to be continued. The PRORIVA was accepted positively and was considered a break from their routine, “boring” activities.

#### High SES communities (Lay people)

Both men and women in the high SES communities said the program improved their knowledge regarding heart disease. Most respondents said the program was “good”, and that there was a “need for continuation”, and a “need for improvement”. Some women said that they tried to participate in the program but the limited citizen participation was reflected in delay or postponement of group activities.

#### High SES communities (health worker)

In contrast to the low SES communities, health workers in high SES communities perceived that their support was in less demand. They did not have the self-confidence to disseminate messages to citizen who might have a higher educational level, Table 4.

Responses to the PRORIVA interventions differed depending on whether participants were residents in a low or high SES community, Table 5. Citizens in low SES communities preferred programs with group orientation and supported community activities. A more individual orientation was preferred by citizens of high SES communities; they did not like the community activities.

**Table 5:** Characteristics of citizen response to the PRORIVA program

**Response to PRORIVA program**	**Low SES community**	**High SES community**
Form of participation	Group orientation	Individual orientation
Attitude to community activities	Supported community activities	Disliked community activities
Perception of the meaningful of program	Health workers thought meaningful	Health workers less welcome
Approach to participating program	Altruistic approach	Individualistic approach

## Discussion

The purpose of this study was to report initial experiences in the development of the PRORIVA program, a community-based intervention to prevent and control CVD. The pilot study was used to describe the process of introducing different intervention components based on participant perspectives, rather than to evaluate outcomes in terms of risk factor changes.

People in the low SES communities reflected their thought that health workers were meaningful for them, while health workers were less welcome in the high SES communities. This is consistent with previous findings where community leaders in low SES communities were expected to play an active role in community interventions while the corresponding role in a high SES community was expected to be more passive [25]. The qualitative study also found that initial motives among low SES community members for program participation were from a feeling of obligation to respond to an action, rather than to prevent disease. In contrast, the motives for high SES community citizens were more oriented to disease prevention, a position that was reflected in their demand for more detailed information. In other words, citizens in the low SES communities had more altruistic motives and participated for the common good. These different initial approaches and preferences for community actions may explain the fact that participation was lower among citizens in high SES communities (Table 2).

Eventually, the motives of citizen in low SES communities became more positive, possibly followed by an increased awareness of the need to prevent CVD, as assumed by Prochaska in the stage-of-change theory to modify behaviour [26]. The different approaches support the idea of “early adopters” among the citizens of high SES communities. This group was more receptive to new information [27]. These finding enhance our understanding of why an intervention has to be designed to address citizens in a low or high SES community, and the designs may differ. Consequently, our study illustrates the importance of community-oriented intervention components when initiating health promotion actions among disadvantaged groups such as citizens in low SES communities.

According to our qualitative data, the target populations initiated their experiments in changing health behaviours by modifying eating and exercise habits, but not smoking habits. The strong peer influence of current smokers made men reluctant to control their own smoking habits, even though their wives expressed an aversion to their husband’s smoking. This finding is in line with a previous study from Java that showed smoking is “a culturally internalized habit” for young men [28].

### Lessons learned from How people responded to PRORIVA

The qualitative part of our study captures existing initiators, the accommodation to people’s preferences, and the determination to match people’s daily activities as encouraging factors for active participation. The results are consistent with earlier findings that physical activity modification is more pronounced if program activities are integrated into daily lives [29]. It is essential that an intervention program optimize the encouraging factors and minimize the barrier factors when performing an intervention program [6,30]. In contrast, strained economic conditions were cited as a frequent barrier to improving eating habits through higher consumption of fruits and vegetables. The intervention program could not influence this problem in the short term. How the participants response to PRORIVA is consistent with findings from a qualitative study in the same location and confirms a low SES lay people’s pattern of preferring collective actions [25].

Especially among citizens in low SES communities, the program was well-tailored to suit local demands, motivate citizens to participate, and able to improve knowledge (among men). The qualitative data illustrated that acceptance of the program was high, as expressed by the pleasure among participants of engaging in PRORIVA activities, and requests for continuation and improvement of the program. The results should however be interpreted with caution. Change takes time, and the period of the pilot study was relatively short. If a similar pilot program is implemented for a longer period of time, further dimensions of behaviour change may be achieved as suggested in “cognitive consistency theory” [31].

### Further opportunities for health promotion

As shown by the qualitative data, women declared that not only did they enjoy the PRORIVA program, but they also actively supported program implementation. This finding is in agreement with previous studies which define women as the caretakers for health [25] and imply women should be considered important entry points for health promotion actions to reach the whole family. To make the best of this potential opportunity, an appropriate strategy must be taken to minimize the extra burden on women, especially in low SES communities, who are already responsible for heavy domestic tasks, as well as frequently being employed.

Maintaining program sustainability is a key issue in a community-empowerment strategy. By involving the community in the problem identification phase so that possible solutions can be discussed, solutions can be implemented and the evaluation process can be done as a joint venture with the community. In this way, we can expect the community members to have a sense of program ownership [11,32].

Recent reviews show that developing countries have a higher CVD burden [2,6,33]. At the same time, their health care systems are not prepared to control chronic diseases, and this includes CVD [34]. Added to this, most studies of CVD prevention and control focus on developed countries, individual-based interventions, and policy level interventions [6]. The essential advantage of this study is that it addresses opportunities and difficulties in a community-based intervention in an urban area within a developing country. Community empowerment is an opportunity at reaching disadvantaged groups in this study.

A critical issue in tackling non-communicable diseases is the need to combine top-down and bottom-up approaches. This is stated implicitly in the policy action document to control non-communicable diseases, adopted at the 2011 United Nations high level meeting on non-communicable diseases [7,35-37]. Top-down actions include reorienting health systems, while bottom-up actions include raising awareness and behaviour modification. This demands participation from the general population as well as non-health sectors such as education, city planning, and the private sector. Thus, a strong multi-sectorial collaboration is a prerequisite [38], and political commitment is required to support all actions [5,35-37,39].

This study provides input on how a bottom-up approach could be applied in developing country. However, this bottom-up approach must be regarded as complementary to the top-down approaches which are already proven to be effective [38,40].

### Limitations

There are several limitations of this study. First, a single-blind trial used in this study may introduce psychological effect of the researcher to expect better response in the intervention group than in the referent group. To minimize this researcher bias, one author and all interviewers who collected data in the pre-test, post-test, free-listing and individual interviews had never been involved in the PRORIVA intervention. Second, the quasi-experimental design may result in uneven distribution of confounders between the intervention and referent groups. To minimize the effect of those confounders, we tried to selected similar sex and age composition of groups before randomly assigned those groups into intervention and referent groups. Careful interpretation should be considered as there might be unknown confounders. However, this dilemma is unavoidable in health promotion intervention field because randomized control trial design would introduce information bias [10]. Third, because the mass media exposure is similar in both interventions and referent areas, it might possibly have introduced contamination bias. However this bias is assumed to be limited as transferring the process of community empowerment is less likely to be delivered through mass media. More intensive interactions are needed for building trust, raising awareness, planning and organizing activities.

## Conclusions

From the citizen point of view, the PRORIVA program increased awareness of the dangers of CVD and encouraged people to change health behaviours. In low SES communities, the intervention was perceived as an attractive activity, while the health workers thought the PRORIVA program was helpful in accomplishing an important task.

The more a health promotion program accommodates to local demands and the greater its appropriateness with regard to the local context, the greater the potential is for acceptance. In this study, the group-oriented community interventions were more appropriate for low SES communities. Individually orientated components were reported to be more appropriate for people in high SES communities.

The paper illustrates that a bottom-up community empowerment strategy can attract the target population to participate in a health promotion program within an urban area in a developing country. By this, it can interact with and complement policy-oriented programs.

## Competing interests

The authors declare that they have no competing interests.

## Authors’ contributions

FD is responsible for coordinating the study, contributed for the formulation of the study design and drafted the manuscript. HS contributed to the formulation of the study and led the quantitative data analysis. UM contributed to the qualitative data analysis. AÖ led the qualitative data analysis. LW led the formulation of the study design and responsible for the supervising of the study. All authors read and commented on drafts of the manuscript and agreed on the final version.

## Authors’ information

Fatwasari Tetradewi – Doctoral student, Epidemiology and Global Health, Department of Public Health and Clinical Medicine, Umeå University, Umeå, Sweden. A local medical doctor and interested in community empowerment to promote health.

Hans Stenlund - Associate professor, researcher in public health science and statistical consultant in epidemiological and medical research projects.

V. Utari Marlinawati - A local anthropologist who works mainly in qualitative data analysis in community research. She was not involved in PRORIVA but performed the qualitative data analysis with the first author.

Ann Öhman – Professor and researcher in public health, specializes in medical sociology, gender studies and qualitative methodology.

Lars Weinehall - Professor in epidemiology and family medicine, researcher in public health, and specializes in designing long term community intervention programs.

## Pre-publication history

The pre-publication history for this paper can be accessed here:

http://www.biomedcentral.com/1471-2458/13/1043/prepub
